# Genome-Wide Discovery and Analysis of Phased Small Interfering RNAs in Chinese Sacred Lotus

**DOI:** 10.1371/journal.pone.0113790

**Published:** 2014-12-03

**Authors:** Yun Zheng, Shengpeng Wang, Ramanjulu Sunkar

**Affiliations:** 1 Faculty of Life Science and Technology, Kunming University of Science and Technology, Kunming, Yunnan, China; 2 Department of Biochemistry and Molecular Biology, Oklahoma State University, Stillwater, Oklahoma, United States of America; Albert Einsten College of Medicine, United States of America

## Abstract

Phased small interfering RNA (phasiRNA) generating loci (briefly as PHAS) in plants are a novel class of genes that are normally regulated by microRNAs (miRNAs). Similar to miRNAs, phasiRNAs encoded by PHAS play important regulatory roles by targeting protein coding transcripts in plant species. We performed a genome-wide discovery of PHAS loci in Chinese sacred lotus and identified a total of 106 PHAS loci. Of these, 47 loci generate 21 nucleotide (nt) phasiRNAs and 59 loci generate 24 nt phasiRNAs, respectively. We have also identified a new putative TAS3 and a putative TAS4 loci in the lotus genome. Our results show that some of the nucleotide-binding, leucine-rich repeat (NB-LRR) disease resistance proteins and MYB transcription factors potentially generate phasiRNAs. Furthermore, our results suggest that some large subunit (LSU) rRNAs can derive putative phasiRNAs, which is potentially resulted from crosstalk between small RNA biogenesis pathways that are employed to process rRNAs and PHAS loci, respectively. Some of the identified phasiRNAs have putative *trans*-targets with less than 4 mismatches, suggesting that the identified PHAS are involved in many different pathways. Finally, the discovery of 24 nt PHAS in lotus suggests that there are 24 nt PHAS in dicots.

## Introduction

Plant genomes encode abundant but diverse populations of small non-coding RNAs, which can be broadly divided into microRNAs (miRNAs) and endogenous small interfering RNAs (siRNAs) [1]. Endogenous siRNAs can be further grouped into several sub-classes such as tasiRNAs, natsiRNAs, heterochromatic siRNAs and phasiRNAs [1], [2]. The role of miRNAs as post-transcriptional regulators is well known [1]–[7]. Among siRNAs, tasiRNAs and natsiRNAs are known to act as guide molecules for post-transcriptional gene regulation, and heterochromatic siRNAs in transcriptional gene silencing, but the role of phasiRNAs in gene regulation is still unclear [8].

The biogenesis of miRNAs consists of several steps. In plants, the primary miRNA transcripts, normally forming hairpin structures, are transcribed by RNA polymerase II (Pol II). The hairpin-like structures are then processed by DICER-LIKE 1 (DCL1) to produce miRNA:miRNA* duplex. Mature miRNAs will be loaded into an RNA-induced silencing complex (RISC), normally with an Argonaute (AGO) protein [5], [6]. Plant miRNAs guide the RISC to their targets on the basis of perfect or nearly perfect complementarities, which normally induce cleavages of their targets at the centers of the complementarities [6].

Similar to miRNAs, *trans*-acting small interfering RNAs (tasiRNAs) are a class of siRNAs that represses their target transcripts at post-transcriptional level. The primary transcripts of tasiRNAs are used to generate double strand RNAs (dsRNAs) by RDR6 (RNA-dependent RNA polymerase 6). The dsRNAs are then cleaved by DCL4 to form phased 21 nt segments [9]–[11] or by DCL5 to form 24 nt phased segments [12], [13]. The precise phasing of tasiRNAs is guided by miRNAs [9] through either two [14] or one [12], [15]–[18] miRNA binding site. Four families of tasiRNA generating loci, named TAS1 to TAS4, have been identified in *Arabidopsis thaliana* [9], [11], [19]. Among these 4 TAS genes, TAS3 is a well conserved gene [14]. In addition to these four typical non-coding genes, accumulating evidences suggest that coding genes, especially PPR [11], [18], [20], NB-LRR disease resistance proteins [18], [21]–[25], MYB transcription factors [18], [26], [27], also generate phased siRNAs. These phased siRNAs do not necessarily function in *trans*, thus are named as phasiRNAs, and their corresponding generating loci are called as PHAS genes by Zhai *et al.*, [21]. Similar to TAS, PHAS from coding genes are also targeted by miRNAs, such as miR161 targeting PPR [11]; miR428 and miR2118 targeting NB-LRR [21], [25] and miR828 targeting MYB transcripts [18], [23], [26].

The genome of the Chinese sacred lotus (*Nelumbo nucifera Gaertn.*), with about 929 Mbp, was recently sequenced [28]. To identify PHAS in this newly sequenced species, we used two small RNA libraries of the leaves and flowers. The phased siRNAs were checked in the genome of the Chinese sacred lotus using a computational approach modified from previous methods in [11], [18], [20], [21]. The potential targets of phasiRNAs from the identified PHAS genes in Chinese sacred lotus were predicted using the HitSensor algorithm [29].

## Materials and Methods

### Data sets

Two small RNA libraries of leaves and flowers of sacred lotus were sequenced using Illumina GAII analyzer, generating 18,505,940 and 29,067,085 reads (a total of 47,573,025 reads) respectively [30]. These two small RNA profiles had been deposited into the NCBI GEO database under the series accession number GSE62217. The genome and cDNA sequences of Chinese sacred lotus (*Nelumbo nucifera Gaertn.*) were downloaded from NCBI GenBank [28]. The sequences of TAS3, TAS4 loci and their derived tasiRNAs were downloaded from the tasiRNAdb [31].

### Computational steps

The unique sequences in the small RNA libraries were mapped to the genome and cDNA sequences of Chinese sacred lotus with SOAP2 [32]. A self-written program was used to scan the genome and cDNA sequences using a window of 210 nt or 240 nt (ten 21 nt or 24 nt) respectively. A two-nucleotide positive offset was used to calculate the positions of siRNAs on the anti-sense strand because the existence of two-nucleotide over-hang at the 3′-end of siRNA duplex [11], [18], [20], [21]. Then a *P*-value was calculated for each of the windows using a modified version of methods in [20],
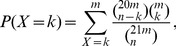
(1)where 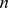 was the number of unique 21 nt (or 24 nt) sRNAs mapped within a window, 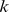 was the number of phased unique 21 nt (or 24 nt) sRNAs within the window, and 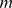 was the number of phases. Similar to previous work [33], 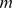 was set to 10 in this study.

And a phase score was calculated for each position of the genome and cDNA sequences using the method in [34]. For a window started at a position with more than three phased unique sRNAs, i.e., when 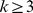,
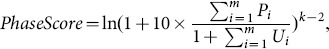
(2)where 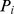 was the number of phased reads at the *i*th phase from the position, 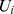 was the number of non-phased reads at the *i*th phase from the position, and 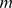 was the number of phases in the window, and 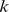 was the number of unique phased siRNAs in the window. 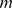 was 10 in this study.

The window with a *P*-value less than 0.05 was extended 100 base pairs at both 5′- and 3′-ends, then the overlapped windows were merged. The *P*-values of the merged windows were used to calculate the false positive rates using the method in [35]. The merged windows with a maximal phase scores of larger than pre-determined threshold and multiple test corrected *P*-values of smaller than 0.05 were reported as PHAS loci. The predicted PHAS were named with its chromosome (or scaffold) and a unique serial number for each chromosome. The neighboring PHAS loci were predicted as PHAS clusters if the distances between individual PHAS loci were smaller than 2,000 base pairs. The phased siRNAs of the predicted PHAS loci were reported as phasiRNAs. The phasiRNAs of a PHAS loci were named by adding siR and a serial number to the name of the PHAS loci.

The miRNA binding sites on PHAS and the targets of predicted phasiRNAs were predicted with the HitSensor algorithm [29]. For 21 nt/22 nt miRNAs and phasiRNAs, targets with less than 4 mismatches were kept for analysis. For 24 nt miRNAs and phasiRNAs, targets with less than 6 mismatches were maintained for analysis.

We combined the annotation of genes of Chinese sacred lotus in [28] with alignment results of predicted PHAS sequences to the NCBI Nucleotide Collection (nr/nt) database and the TIGR Repeat database [36].

The phylogenetic trees of the predicted TAS3, TAS4 loci and their derived tasiRNAs were constructed with the Bootstrap Neighboring-Joining algorithm implemented in ClustalX (version 2.1) [37] and visualized with TreeView [38].

## Results and Discussion

### 21 nt PHAS loci in Chinese sacred locus

We predicted PHAS loci by using the alignments of small RNA sequencing libraries to the genome and cDNA database respectively. The predicted PHAS loci were combined and merged if necessary. As listed in Table 1, we totally identified 16 and 7 loci corresponding to 21 nt and 24 nt PHAS loci, respectively, when using a phase score threshold of 10 (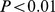, as shown in Figure S1a). After relaxing the phase score threshold to 5 (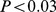, see Figure S1a), we identified 31 additional 21 nt PHAS loci (in Table S1), three of which are shown in Table 1.

**Table 1:** Some identified PHAS loci of Chinese sacred lotus.

PHAS_ID	Start	End	TR	PR	*P*-value	FDR	P.S.	Locus Annotation	Ref.
*21 nt*									
scaffold_106_1	375,169	376,010	94	9	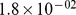	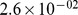	15.6	putative TAS3a	[9], [11], [30]
scaffold_107_1	1,397,214	1,397,907	87	11	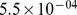	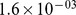	18.1	unknown	
scaffold_10_1	1,954,273	1,954,966	88	9	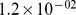	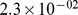	12.2	putative TAS3c	[9], [11], [30]
scaffold_131_1	70,673	71,429	97	11	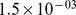	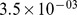	26.1	Putative disease resistance protein RGA3	[21]
scaffold_149_1	773,189	773,819	156	12	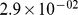	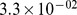	10.4	LSU-rRNA	
scaffold_170_1	166,669	167,980	61	10	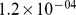	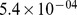	29.2	unknown	
scaffold_326_1	253,709	254,360	170	13	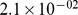	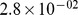	12.9	LSU-rRNA	
scaffold_326_2	254,166	254,838	58	6	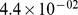	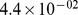	11.5	LSU-rRNA	
scaffold_326_3	324,761	325,370	162	12	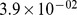	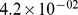	21.2	LSU-rRNA	
sf_39_1	2,038,471	2,039,238	143	12	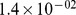	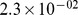	15.9	putative TAS4	[19]
scaffold_65_1	675,292	676,027	73	10	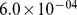	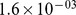	27.0	putative TAS3b	[9], [11], [30]
scaffold_87_1	119,206	120,004	33	5	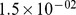	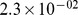	13.5	putative NB-LRR disease resistance protein	[21]
sf_88_1	539,406	540,015	101	9	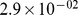	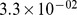	13.0	LSU-rRNA	
scaffold_8_1	9,281,139	9,281,769	12	6	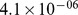	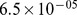	10.8	Putative disease resistance protein RPP13	[21]
scaffold_8_2	9,353,049	9,353,721	90	12	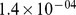	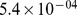	13.6	Protein of unknown function	
scaffold_90_1	1,885,970	1,886,621	25	7	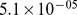	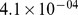	10.8	putative Transcription factor MYB90	[26]
PHAS_sf_173_1	496,493	497,186	45	7	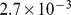	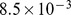	7.0	putative MYB transcription factor	[26]
PHAS_sf_21_1	1,961,563	1,962,329	81	8	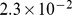	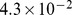	7.8	putative WER, MYB transcription factor	[26]
PHAS_sf_122_1	581,162	581,771	4	4	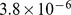	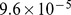	5.6	putative NB-LRR disease resistance protein	[21]
*24 nt*									
scaffold_24_1	6,030,639	6,031,302	82	7	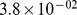	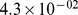	10.0	intergenic region	
scaffold_252_1	376,585	377,296	49	5	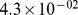	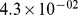	12.8	intergenic region	
scaffold_287_1	359,861	360,500	20	5	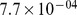	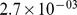	11.2	unknown gene	
scaffold_39_1	2,038,529	2,039,168	81	7	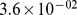	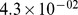	10.2	overlapped with sf_39_1	
scaffold_42_1	2,581,628	2,582,459	29	4	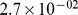	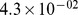	12.7	expressed sequence match	
scaffold_5_1	3,813,044	3,813,827	7	4	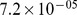	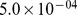	10.1	intron of a protein of unknown function	
scaffold_88_1	1,770,018	1,770,657	96	8	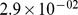	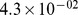	15.1	unknown gene	

The Start and End column list the start and end positions of the predicted PHAS loci in the scaffold. The TR and PR column list the total number of unique siRNAs and the number of phased unique siRNAs, respectively. The *P*-value and FDR column list the *P*-values evaluated with Equation 1 and the false discovery ratio using method in [35]. The P.S. column lists the phase scores calculated using Equation 2. The Ref. column lists related literature of the predicted PHAS loci.

**Figure 1:**
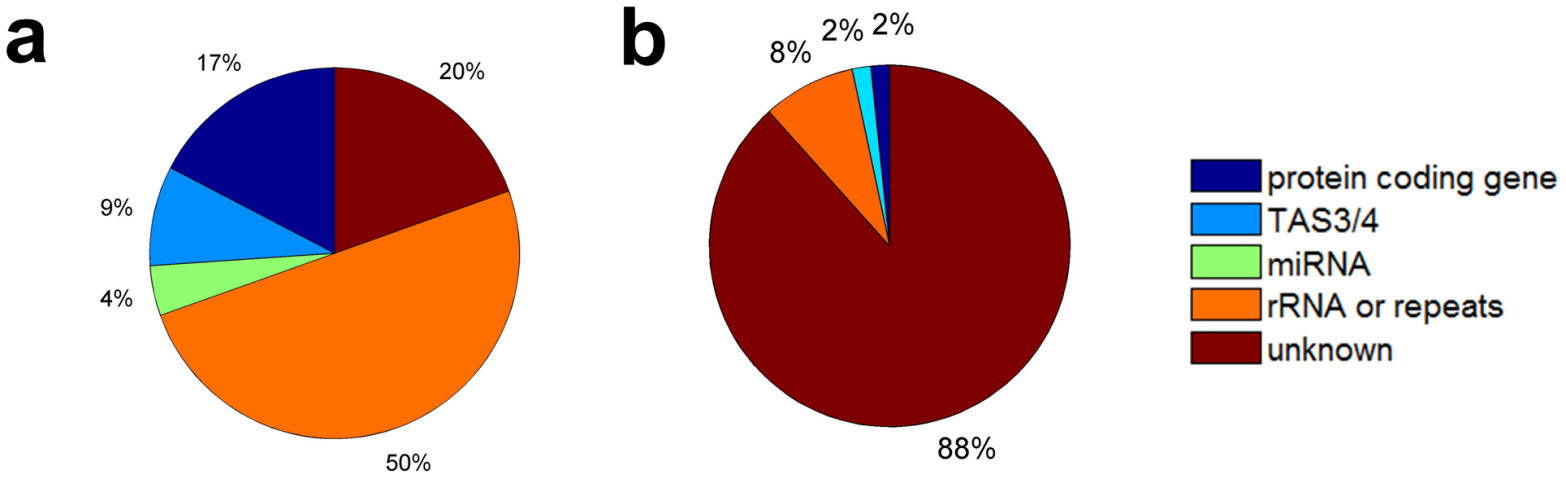
The category of predicted PHAS loci. (a) The category of 21 nt PHAS loci. (b) The category of 24 nt PHAS loci.

In addition to the annotation from [28], we aligned the predicted PHAS to the NCBI Nucleotide Collection (nr/nt) database and the TIGR Plant Repeat database to refine putative annotation of the predicted PHAS loci (details are given in Table S1). Furthermore, as miRNAs are critical in generation of phasiRNAs, we predicted miRNA complementary sites on these PHAS loci on both strands. Based on the miRNA complementary sites, three loci are classified as TAS3 and one locus is classified as TAS4, which will be discussed below. As shown in Figure 1a, the largest category, 50%, of 21 nt PHAS loci are rRNA or repeats. There are eight (17%) 21 nt PHAS overlapped to protein coding genes. Four (9%) 21 nt PHAS loci are TAS3 and TAS4 loci, and two (4%) 21 nt PHAS are pre-miRNA159 loci.

**Figure 2:**
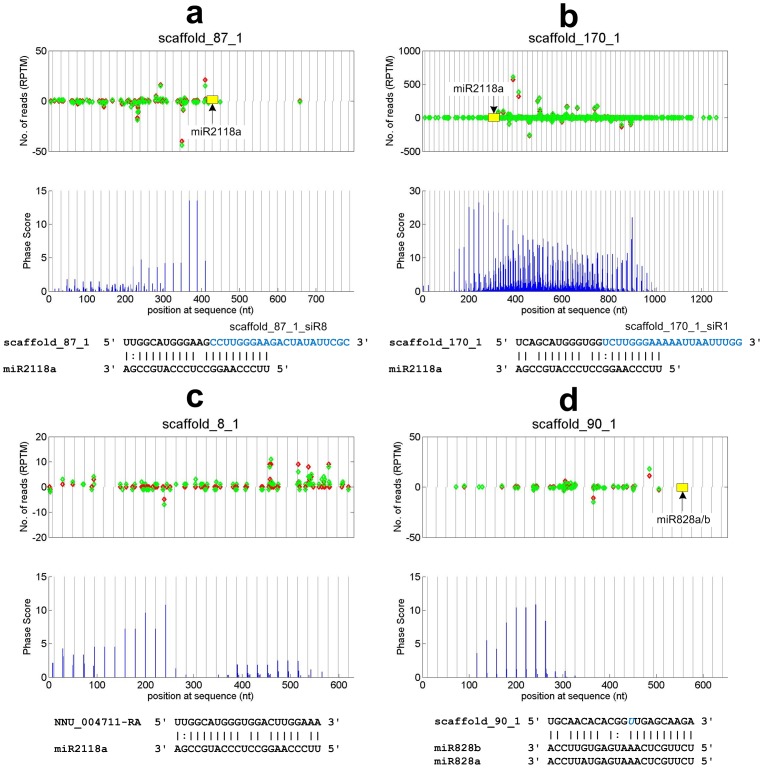
The read distributions and phase scores of some predicted PHAS loci from NB-LRR disease resistance proteins and a MYB transcription factor. The red and green diamonds represent the number of 21 nt reads, vertical axis, that appeared at the position of the PHAS loci, horizontal axis, in the flower and leaf small RNA libraries, respectively. The vertical gray lines with distances of 21 nt are the phased positions from the position with highest phase scores of the PHAS loci. The yellow boxes in the read distribution panel represent the miRNA complementary sites. Sites pointed by miRNAs from above and under zero read line means miRNAs complement to the plus and minus strand of the predicted PHAS loci, respectively. The predicted miR2118a complementary sites are shown below the phase score panel. (a) to (c) Three potential PHAS loci, scaffold_87_1, scaffold_170_1 and scaffold_8_1, from NB-LRR disease resistance proteins. The blue sequences in the complementary sites of (a) and (b) are one of the phasiRNAs from the PHAS loci. The miR2118a site in Part c is at 176 nt 5′ side (upstream) of the PHAS locus scaffold_8_1. (d) scaffold_90_1, from an MYB90 transcription factor locus.

Previous studies found that miR1507, miR1509 and miR2118 families trigger several PHAS loci, especially the NB-LRR disease resistance genes that possess one complementary site to miR2118a, a 22 nt miRNA [21]. Our analysis also predicted miR2118 complementary sites on three predicted PHAS loci, scaffold_87_1 (Figure 2a), PHAS_sf_122_1, and scaffold_170_1 (Figure 2b), from which phased siRNAs are originated, suggesting these loci are NB-LRR family members. Furthermore, after aligning their sequences to the NCBI nr/nt database, PHAS_sf_122_1 and scaffold_87_1 are putative disease resistance proteins (with E-values of 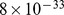 and 0.002, respectively, see Table S1). Another locus, scaffold_8_1 overlapping with a putative disease resistance RPP13-like protein 1 (NNU_004711-RA) also has a miR2118 site in the upstream region (Figure 2c). However, there is no miR2118 site around scaffold_131_1, which overlaps with a putative disease resistance protein RGA3 (see Table 1).

miR828 targeted MYB genes can generate phasiRNAs in apple [26]. Three PHAS loci scaffold_90_1, PHAS_sf_173_1 and PHAS_sf_21_1 possess complementary sites to miR828 in sacred lotus (see Figure 2d, Figure S2a and Figure S2b). scaffold_90_1 and PHAS_sf_21_1 overlap with two MYB transcription factors (NNU_018790 and NNU_008785 respectively). PHAS_sf_173_1, shown in Figure S2a and S2c, is a potential MYB transcription factor after aligning its sequence to the NCBI nr/nt database (with an E-value of 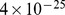, see Table S1).

As listed in Table S1, the 21 nt PHAS loci also form three additional PHAS clusters in addition to scaffold_326_1/2 in Table 1 and Figure 5a to b.

We do not find TAS1 and TAS2 loci in Chinese sacred locus, probably because these two TAS families are less conserved than TAS3 [8].

A substantial number of predicted 21 nt PHAS loci, 20%, are located in regions of unknown genes or intergenic regions, as shown in Figure 1a. They also did not matched to known genes in NCBI nr/nt database and known repeat elements in TIGR Repeat database. Further research is needed to clarify whether these are also PHAS loci or not in sacred lotus.

### Discovery of yet another TAS3 locus in Chinese sacred locus

**Figure 3:**
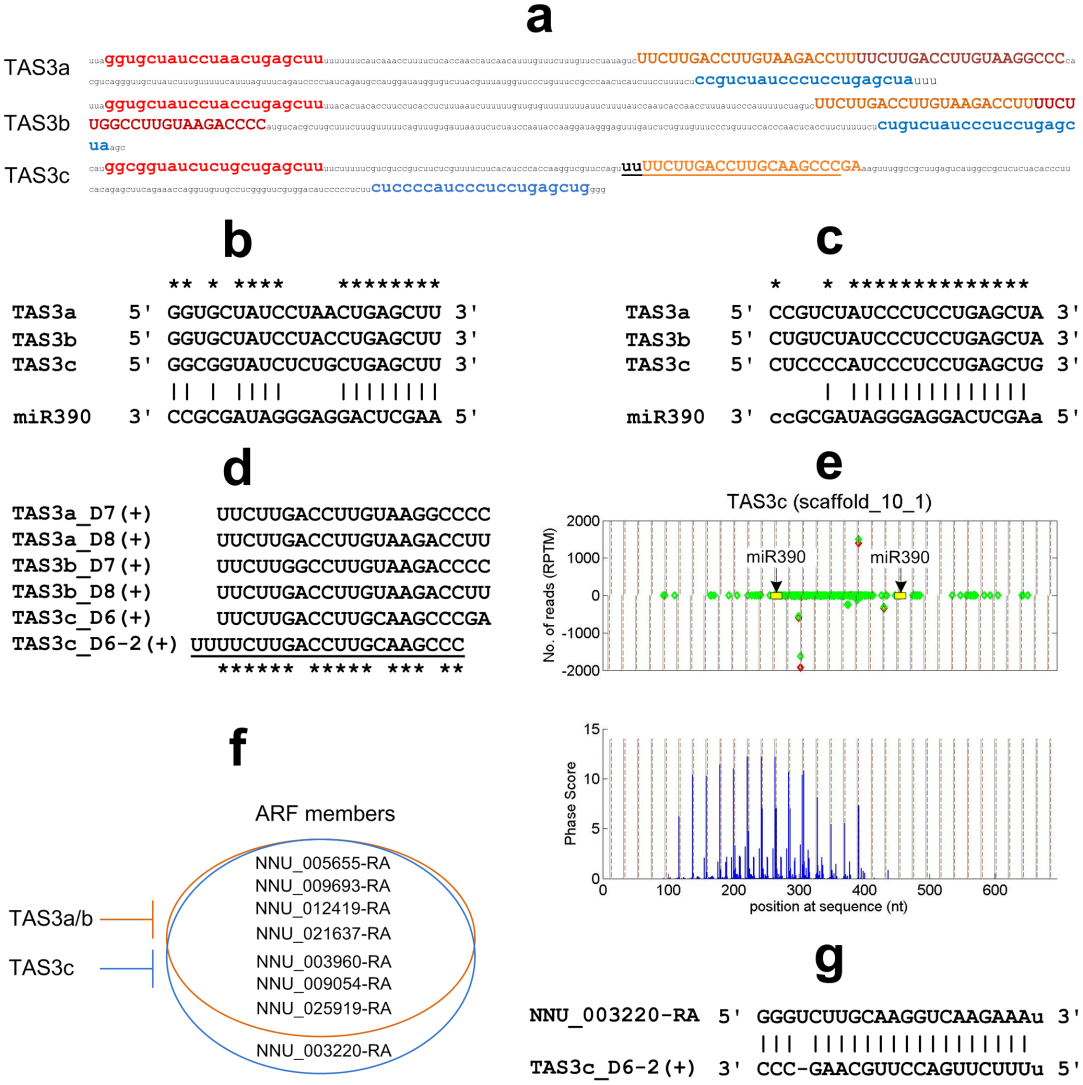
Putative TAS3c locus and the predicted targets of tasiRNAs in Chinese sacred lotus. (a) The sequences of the three putative TAS3 loci in Chinese sacred lotus. The red and blue regions are 5′ and 3′ miR390 complementary sites, respectively. The regions of upper case nucleotides are mature tasiRNAs that target ARF family members, or tasiARFs. The underlined region of TAS3c are a phased siRNA TAS3_D6-2(+) with position 12. (b) The 5′ miR390 binding sites on TAS3 transcripts. Only the commonly aligned nucleotides are aligned to miR390. (c) The 3′ miR390 binding sites on TAS3 transcripts. (d) The mature tasiRNAs that target ARF family members derived from TAS3a/b/c loci. (e) The reads distribution and phase score of TAS3c. Legend are the same as those in Figure 2. The vertical dashed lines with distances of 21 nt are phase positions that start from position 10 of the 3′ miR390 site. (f) The predicted targets of tasiRNAs. The yellow and blue circle includes 7 and 8 ARF family members that are targeted by TAS3a/b and TAC3c derived tasiRNAs, respectively. (g) The complementary site of TAS3_D6-2(+) and an ARF family member, NNU_003220-RA.

Among the sixteen 21 nt PHAS loci, three are TAS3 loci, as shown in Figure 3a. Two of them were identified in our previous study [30]. The typical 5′ and 3′ miR390 complementary sites around the phased siRNA regions of these TAS3 loci are given in Figure 3b and c, respectively. TAS3c is unique since (i) its phase starts from two positions (position 10 and 12) of the 3′ miR390 complementary site; (ii) TAS3 only encode one conserved tasiARF (tasiRNAs that target ARF family members [14]), as shown in Figure 3a. The conserved tasiRNA originated from position 12 has a two nucleotide shift, named as TAS3c_D6-2(+) (in Figure 3d). The most abundant siRNA from the whole TAS3 gene is also in phase with position 12, as shown in Figure 3e. The targets of the conserved tasiRNAs include 8 ARF family members. One of these ARF genes is targeted by TAS3c siRNAs with much better complementarities, especially by TAS3c_D6-2(+) (as shown in Figure 3g). These results suggest that the two nucleotide shift in the phase of TAS3 may be beneficial to its targeting to the additional ARF member (NNU_003220-RA). TAS3c may be a shorter variant of TAS3 which only encodes one tasiRNA and both the 5′ and 3′ miR390 sites of this variant are cleavable as mentioned in [8], [26]. As shown in Figure 3b, the 5′ miR390 site on TAS3c has more matched nucleotides at positions 10, 11 and 19 than TAS3a and TAS3b, suggesting this site is cleavable.

We performed conservation analysis for the TAS3 loci and their derived tasiRNAs to show their relations to TAS3 genes in other species as shown in Figure S3. The identified TAS3a and TAS3b have a close relation, but TAS3c is far from the cluster of TAS3a and TAS3b (Figure S3a), which is in accordance with the unique features of TAS3c discussed above. TAS3c derived tasiRNA is also distant from TAS3a and TAS3b derived tasiRNAs (Figure S3b and S3c).

### Discovery of TAS4 locus

**Figure 4:**
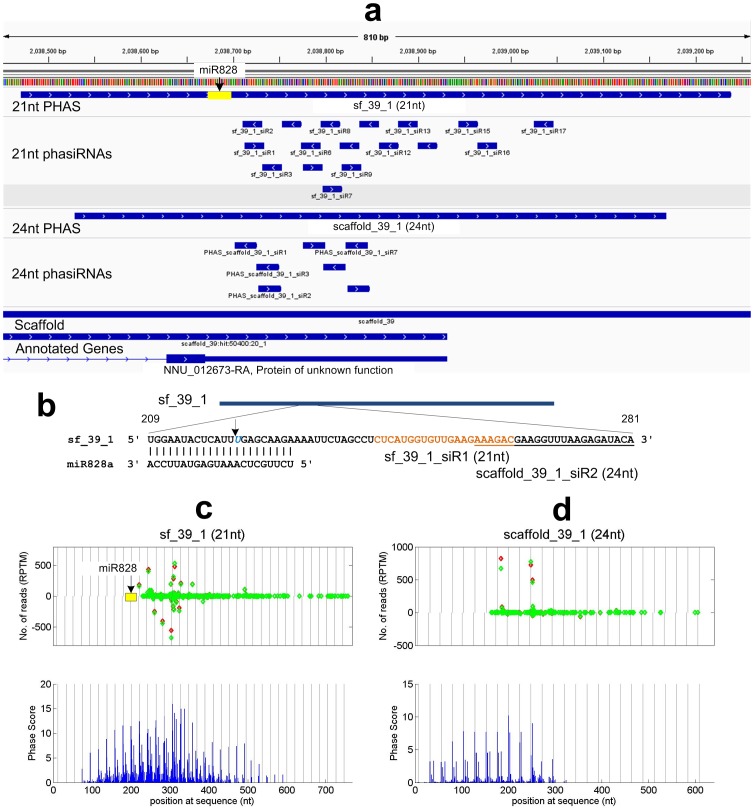
Putative TAS4 (sf_39_1, NNU_012673-RA) derives both 21 nt and 24 nt phasiRNAs. (a) The schematic view of the predicted 21 nt and 24 nt putative TAS4 and its derived phasiRNAs, as well as annotated genes. (b) The miR828 site on putative TAS4 (sf_39_1). The yellow and underlined region represent the 21 nt and 24 nt that are nearest to the miR828 site. The position pointed by an arrow is the expected phase start position that is triggered by miR828. (c) and (d) The read distribution and phase score of the 21 nt and 24 nt PHAS loci predicted. The legend are the same as those in Figure 2. The distances between vertical gray lines in Part c and d are 21 nt and 24 nt, respectively.

As shown in Figure 4a and b, one 21 nt PHAS locus (sf_39_1) has a typical miR828 complementary site of TAS4 [19] at the 5′ side of the phased region. This locus is annotated as a protein of unknown function, NNU_012673-RA [28], with two exons. Together with these results, conservation analysis suggests that this locus is a conserved TAS4 gene (see Figure S4a). The transcription region of this locus is longer than the exon regions of NNU_012673-RA because there are some phased and non-phased siRNAs beyond the 3′ end of the second exon of NNU_012673-RA, as shown in Figure 4a and c. One of the siRNAs, sf_39_1_siR4, derived from sf_39_1 is highly conserved to TAS4-siR81(−) reported in *Arabidopsis thaliana* [19] and other species [31] (see Figure S4b and S4c). Thus, it is named as TAS4-siR81(−) too. It is interesting that this locus also produces phased 24 nt siRNAs as shown in Figure 4b and d. This suggests that TAS4 transcripts of sacred lotus can be processed by both DCL4 and DCL5 (also named as DCL3b) [8], [12], [13] to produce both 21 nt and 24 nt phasiRNAs, respectively.

**Figure 5:**
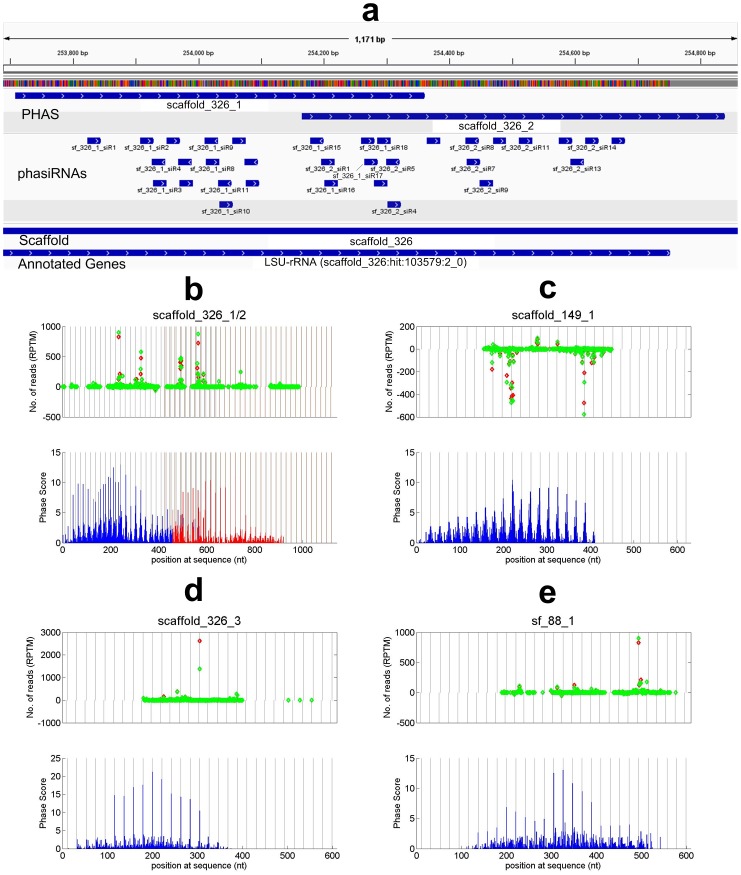
Some LSU-rRNA loci that generate putative phasiRNAs. (a) A schematic view of phasiRNAs, and annotated genes around two predicted PHAS loci scaffold_326_1 and scaffold_326_2. (b) to (e) The read distributions and phase scores of scaffold_326_1/2, scaffold_149_1, scaffold_326_3 and sf_88_1, respectively. The legend are the same as those in Figure 2. In the lower panel of Part b, the blue and red bars represent the phase scores of scaffold_326_1 and scaffold_326_2, respectively.

### LSU-rRNA derived 21 nt phased siRNAs

Five 21 nt PHAS loci are annotated as LSU-rRNA (Table 1) and two of them overlap with each other (Figure 5a and b). It was observed that rRNAs could generate small RNAs [39]–[41] which is dependent on two RNA-dependent RNA polymerases, RDR2 and RDR6, and DCL2/3/4 [40]. Since phasiRNA biogenesis also requires RDR6 and DCL4 [8], the pathways for generating small RNAs from PHAS and rRNA loci share some key protein components with each other. Our results suggest that some LSU-rRNAs are processed through the phasiRNA biogenesis pathway to produce 21 nt phasiRNAs.

### 24 nt PHAS loci

**Figure 6:**
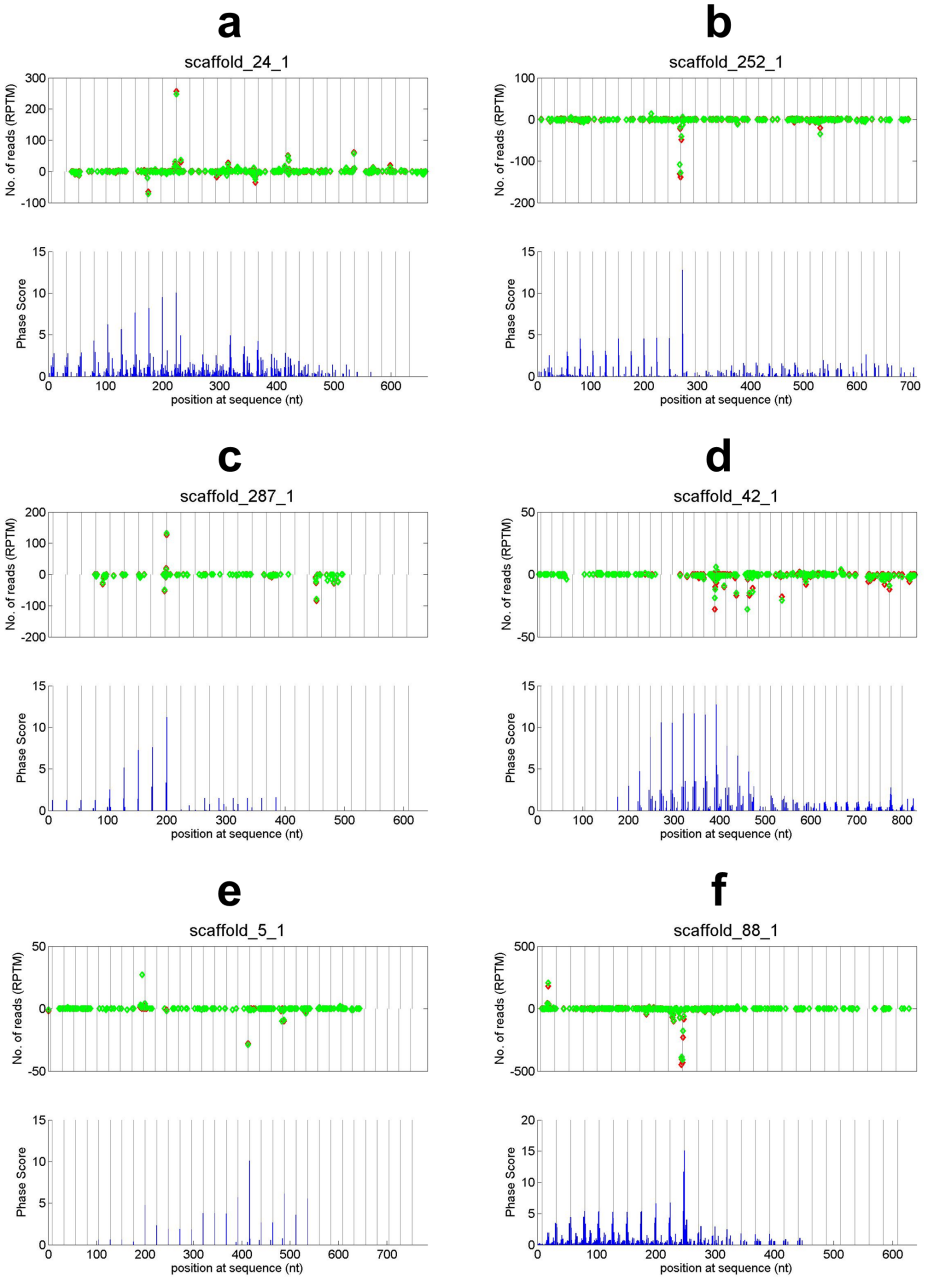
The read distribution and phase scores of some predicted 24 nt PHAS loci. The legend are the same as those in Figure 2 except that the diamonds represent 24 nt reads and the distances between vertical gray lines are 24 nt. (a) scaffold_24_1. (b) scaffold_252_1. (c) scaffold_287_1. (d) scaffold_42_1. (e) scaffold_5_1. (f) scaffold_88_1.

We identified 7 PHAS loci that generate 24 nt long phasiRNAs using a phase score threshold of 10 (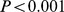, see Figure S1b), as shown in Table 1 and Figure 6. Fifty two additional 24 nt PHAS loci are predicted when using 5 as the threshold of phase score (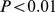, see Figure S1b) as listed in Table S1. The 24 nt PHAS loci form 11 PHAS clusters as shown in Table S1. Similarly, we also aligned the 24 nt PHAS to the NCBI nr/nt database and the TIGR Plant Repeat database to obtain putative annotation of these PHAS loci (details are given in Table S1). As shown in Figure 1b, an overwhelming portion, 88%, of 24 nt PHAS is unknown genes or elements because they have no matches to the known genes and known repeat elements. Compared to 21 nt PHAS, more researches are needed to clarify these PHAS with unclear annotation. Five (8%) 24 nt PHAS are matched to rRNA or repeats. One locus, scaffold_39_1, is TAS4 as mentioned above. Unlike the 21 nt phasiRNAs being initiated by miR828, no significant miRNA complementary sites are predicted to originate 24 nt phasiRNAs at scaffold_39_1, as well as other 24 nt PHAS loci, although existing studies indicate that miR2275 triggers the generation of 24 nt phasiRNAs in rice [12], [13]. Only one 24 nt PHAS is aligned to a hypothetical protein (with an E-value of 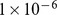).

24 nt PHAS loci have mainly been reported in monocots [8], [12], [13]. Together with the 24 nt PHAS loci reported in grapevine [33], our results suggest that dicots may also have 24 nt PHAS loci, although their biogenesis processes are still to be clarified.

### 21 nt phasiRNAs and their putative targets

The first sixteen 21 nt PHAS loci (Table 1) totally derived 224 phasiRNAs, as shown in Table S2. The abundance of these phasiRNAs is similar in the flower and leaf small RNA libraries, suggesting that these phasiRNAs have similar functions in these two tissues. The most differently expressed phasiRNA with more than 100 RPTM in both libraries is an siRNA derived from an LSU-rRNA (scaffold_326_3), as shown in Figure 5d. Its abundance has dropped from 2600 RPTM in flower to 1300 RPTM in leaf. It needs further investigation to clarify whether such differential abundance has any significance or not.

Several phasiRNAs have perfect alignments to the transcripts on their antisense strands, which are regarded as *cis*-targets. Targets from other loci are regarded as *trans*-targets. Some of the *trans*-targets with the least numbers of mismatches in their complementary sites are listed in Table 2 except the well-known ARF family members targeted by TAS3 derived siRNAs (which have been shown in Figure 3f). As shown in Table 1 and Figures S4b/S4c, a conserved TAS4 derived phasiRNA (sf_39_1_siR4 or TAS4-siR81(−)) targets an MYB transcription factor (NNU_018790). And in Table S3, sf_39_1 derived phasiRNAs target five other MYB transcription factors, two of which are targeted by sf_39_1_siR4. In comparison, MYB transcription factors are also targeted by TAS4 derived siRNAs in Arabidopsis [19], [26], [27], [42]. PhasiRNAs derived from putative NB-LRR genes scaffold_170_1 and scaffold_87_1 potentially target as many as 49 and 44 different members of the NB-LRR disease resistance protein family (see Table 2 and Table S3), respectively. PhasiRNAs derived from another putative NB-LRR gene (NNU_004711-RA), scaffold_8_1, only has one putative *trans*-target in the NB-LRR disease resistance protein family (NNU_025710-RA). Similarly, phasiRNAs derived from a putative MYB gene (scaffold_90_1) potentially target 9 other MYB family members in *trans* (see Table S3). These results are consistent with those reported in other species [8], [21], [26], and similar to siRNAs derived from PPR genes [11], [43].

**Table 2:** Some predicted targets of phasiRNAs derived from the predicted PHAS loci.

PhasiRNA	Target ID	M.	Target Description
*21 nt*			
sf_39_1_siR4	NNU_018790	0	Similar to MYB90 Transcription factor MYB90
sf_8_2_siR11	NNU_017525	1	Protein of unknown function
sf_8_2_siR13	NNU_017525	1	Protein of unknown function
sf_8_2_siR11	NNU_022433	1	Protein of unknown function
sf_170_1_siR16	NNU_021230	1	Similar to RGA3 Putative disease resistance protein RGA3
sf_170_1_siR27	NNU_021230	1	Similar to RGA3 Putative disease resistance protein RGA3
sf_87_1_siR3	NNU_025043	1	Similar to RGA4 Putative disease resistance protein RGA4
sf_8_2_siR11	NNU_020674	1	Similar to At4g18490 Uncharacterized protein
sf_170_1_siR16	NNU_004711	1	Similar to RPPL1 Putative disease resistance
sf_90_1_siR5	NNU_018789	1	Similar to MYB114 Transcription factor MYB114
sf_90_1_siR4	NNU_018789	1	Similar to MYB114 Transcription factor MYB114
sf_107_1_siR3	NNU_023201	1.5	Protein of unknown function
sf_8_2_siR14	NNU_022433	1.5	Protein of unknown function
sf_87_1_siR3	NNU_018068	1.5	Similar to RPPL1 Putative disease resistance
sf_170_1_siR6	NNU_021731	1.5	Similar to ABCG15 ABC transporter G family member 15
sf_170_1_siR17	NNU_000750	1.5	Similar to At5g15080 Probable receptor-like protein kinase
sf_107_1_siR1	NNU_006211	1.5	Similar to EX1 Protein EXECUTER 1, chloroplastic
sf_170_1_siR27	NNU_002459	1.5	Similar to RGA4 Putative disease resistance protein RGA4
sf_87_1_siR3	NNU_002459	1.5	Similar to RGA4 Putative disease resistance protein RGA4
sf_107_1_siR1	NNU_005527	1.5	Similar to At3g12360 Ankyrin repeat-containing protein
sf_87_1_siR3	NNU_018017	1.5	Similar to RPPL1 Putative disease resistance
sf_170_1_siR25	NNU_012692	1.5	Similar to RGA3 Putative disease resistance protein RGA3
sf_90_1_siR4	NNU_018784	1.5	Protein of unknown function
sf_8_2_siR11	NNU_004714	1.5	Protein of unknown function
sf_90_1_siR5	NNU_018786	1.5	Similar to MYB90 Transcription factor MYB90
sf_90_1_siR4	NNU_018786	1.5	Similar to MYB90 Transcription factor MYB90
sf_8_1_siR6	NNU_007200	1.5	Similar to At4g15470 BI1-like protein
sf_106_1_siR10	NNU_011742	1.5	Similar to GATA18 GATA transcription factor 18
sf_39_1_siR8	NNU_008226	1.5	Similar to SLC4A1AP Kanadaptin (Homo sapiens)
sf_10_1_siR10	NNU_014786	1.5	Similar to UBP22 Ubiquitin carboxyl-terminal hydrolase 22
sf_8_2_siR13	NNU_004714	1.5	Protein of unknown function
*24 nt*			
sfd_88_1_siR7	NNU_012891	2.0	Similar to slr0992 Putative tRNA methyltransferase
sfd_5_1_siR8	NNU_013967	2.0	Similar to PDR12 Pleiotropic drug resistance protein 12
sfd_24_1_siR3	NNU_016850	2.5	Similar to DNAJB13 DnaJ homolog subfamily B member 13
sfd_88_1_siR6	NNU_012697	2.5	Similar to At1g68650 GDT1-like protein 5
sfd_5_1_siR8	NNU_016474	2.5	Similar to TIFY6B Protein TIFY 6B
sfd_39_1_siR3	NNU_003001	2.5	Similar to MYB315 Myb-related protein 315
sfd_88_1_siR7	NNU_010637	2.5	Similar to ABP19A Auxin-binding protein ABP19a
sfd_88_1_siR7	NNU_011269	2.5	Protein of unknown function
sfd_88_1_siR7	NNU_005826	2.5	Protein of unknown function
sfd_39_1_siR3	NNU_017703	2.5	Similar to arfA ADP-ribosylation factor 1
sfd_88_1_siR8	NNU_007725	2.5	Similar to WRKY33 Probable WRKY transcription factor 33
sfd_88_1_siR7	NNU_010742	2.5	Similar to NPR1 Regulatory protein NPR1
sfd_88_1_siR7	NNU_009141	2.5	Similar to TPR2 Topless-related protein 2

The M. column is the number of mismatches of the miRNA complementary sites. The names of the 21 nt and 24 nt phasiRNAs have been abbreviated to start with sf_ and sfd_, respectively.

As shown in Table 2, phasiRNAs from the predicted PHAS loci also target many other genes with or without known functions. The biological relevance of these predicted targets still needs additional studies.

### 24 nt phasiRNAs and their putative targets

The seven 24 nt PHAS loci in Table 1 totally generate sixty six 24 nt long phasiRNAs (in Table S2). Similar to 21 nt phasiRNAs, these 24 nt phasiRNAs have similar abundance in the flower and leaf sequencing libraries (see Table S2). Some of the predicted *trans*-targets of these 24 nt phasiRNAs are given in Table 2 and a full list is given in Table S3. As shown in Table 2, sfd_39_1_siR3 potentially targets an Myb-related protein 315 (NNU_003001) with only 2.5 mismatches in a 24 nt complementary site. As mentioned earlier, TAS4a-siR81(−) (i.e., sf_39_1_siR4) potentially targets an MYB transcription factor (NNU_018790), which is a conserved mechanism [19], [26], [27], [42]. sfd_39_1_siR3 is 24nt long and 3 nt downstream of TAS4a-siR81(−), which is potentially beneficial to its complementarity to NNU_003001 because the 21 nt sequence immediately downstream of TAS4a-siR81(−) has 5.5 mismatches to the same complementary site on NNU_003001. These results suggest that the potential 24 nt phasiRNAs derived from TAS4a (sf_39_1), as shown in Figure 4, might be produced as alternative small RNA guiders to repress a set of MYB transcription factors other than those repressed by the conserved 21 nt TAS4a-siR81(−). Another 24 nt phasiRNA sfd_88_1_siR7 has 6 targets with less than 3 mismatches (see Table 2), suggesting it is biologically relevant. However, more studies are necessary to verify whether these 24 nt phasiRNAs are really functional and have *trans*-targets.

## Conclusion

Through genome-wide analysis of two small RNA sequencing libraries, we totally identified 23 putative PHAS loci from Chinese sacred lotus, and 83 additional PHAS loci were further identified with a smaller phase score threshold, including a new putative TAS3 and a putative TAS4 loci. Our results show that the predicted TAS4 loci derives both 21 nt and 24 nt phasiRNAs, suggesting that both DCL4 and DCL5 are involved in the biogenesis of phasiRNAs from TAS4. Several PHAS loci are from NB-LRR and MYB loci, which is consistent with existing results in other species. Existing studies reported that small RNAs are derived from PHAS and rRNA through different pathways with shared protein components, RDR6 and DCL4. In this study, we found that some LSU-rRNA could generate phasiRNAs, suggesting that some rRNAs are processed by the PHAS siRNA biogenesis pathway. However their biogenesis still needs further studies. The identification of 24 nt PHAS loci in Chinese sacred lotus, as well as similar results reported in grapevine [33], suggests that dicots may encode 24 nt PHAS.

## Supporting Information

Figure S1 **The histogram of Phase Scores.** (a) The histogram of Phase Scores of 21 nt PHAS loci. (b) The histogram of Phase Scores of 24 nt PHAS loci. The percentage values above 5 and 10 are percentage of PHAS loci with Phase Scores larger than or equal to 5 and 10, respectively.
(TIF)

Figure S2 **Two PHAS loci, PHAS_sf_173_1 and PHAS_sf_21_1, from putative MYB transcription factors.** (a) - (b) The upper and lower panels show the read distribution and phase scores. The red and green diamonds represent the number of 21 nt reads, vertical axis, that appeared at the position of the PHAS loci, horizontal axis, in the flower and leaf small RNA libraries, respectively. The yellow boxes in the read distribution panel represent the miRNA complementary sites. Sites pointed by miRNAs from above and under zero read line means miRNAs complement to the plus and minus strand of the predicted PHAS loci, respectively. (c) - (d) The miR828b complementary site on PHAS_sf_173_1 and miR828a complementary site on PHAS_sf_21_1, respectively. The italic characters are the expected cleavage sites induced by miR828, i.e., the start positions of the phasiRNAs. The brown regions are phasiRNAs that appear in our sequencing libraries, PHAS_sf_173_1_siR3 (in Part c) and PHAS_sf_21_1_siR8 (in Part d), which are 126 nt (six 21 nt phases) and 21 nt (one 21 nt phase) downstream of the expected cleavage sites induced by miR828, respectively.
(TIF)

Figure S3 **The conservation analysis of TAS3 loci and derived tasiRNAs in Chinese sacred lotus and other species.** (a) The phylogenetic tree of TAS3. (b) The phylogenetic tree of TAS3 derived tasiRNAs. (c) The multiple sequence alignment of TAS3 derived tasiRNAs generated with ClustalX (version 2.1) [37]. The sequences of TAS3 loci and derived tasiRNAs were used to construct the phylogenetic trees with the Bootstrap Neighbor-Joining algorithm implemented in ClustalX (version 2.1). Then, the trees were visualized with TreeView [38]. The numbers in the trees are bootstrap values greater than 500 (50%). The lower case letters at the beginnings of the names of TAS3 and tasiRNAs stand for the species, i.e., at (*Arabidopsis thaliana*), bn (*Brassica napus*), cl (*Cunninghamia lanceolata*), cm (*Cucumis melo*), gm (*Glycine max*), lj (*Lotus japonicus*), md (*Malus domestica*), mt (*Medicago truncatula*), nn (*Nelumbo nucifera (Gaertn)*), nt (*Nicotiana tabacum*), oe (*Olea europaea*), os (*Oryza sativa*), ppa (*Physcomitrella patens*), ppe (*Prunus persica*), sl (*Solanum lycopersicum*), ta (*Triticum aestivum*), vv (*Vitis vinifera*), and zm (*Zea mays*). nnTAS3a, nnTAS3b and nnTAS3c are scaffold_106_1, scaffold_65_1, and scaffold_10_1, respectively. The tasiRNAs of TAS3a/b/c in Chinese sacred lotus are given in Figure 3d.
(TIF)

Figure S4 **The conservation analysis of TAS4 loci and derived tasiRNAs in Chinese sacred lotus and other species.** (a) The phylogenetic tree of TAS4. (b) The phylogenetic tree of TAS4 derived tasiRNAs. (c) The multiple sequence alignment of TAS4 derived tasiRNAs. The legend are the same as those of Figure S3. The lower case letters at the beginnings of the names of TAS3 and tasiRNAs stand for the species, i.e., at (*Arabidopsis thaliana*), md (*Malus domestica*), nn (*Nelumbo nucifera (Gaertn)*), ppe (*Prunus persica*), and vv (*Vitis vinifera*). nnTAS4 is sf_39_1 and nnTAS4-siR81(−) is sf_39_1_siR4.
(TIF)

Table S1 **The predicted PHAS loci in Chinese sacred lotus.** The loci of ID started with scaffold_ and sf_ are predicted with a phase score threshold of 10, and loci of id started with PHAS_ are predicted with a phase score threshold of 5. The loci from the same PHAS cluster are marked with the same cluster ID in the Cluster column.
(XLSX)

Table S2 **The phasiRNAs derived from PHAS with a phase score of ≥10.** The 21 nt and 24 nt phasiRNAs are shown in the first and the second sheet, respectively.
(XLSX)

Table S3 **The predicted targets of phasiRNAs in Chinese sacred lotus.** The targets of 21 nt and 24 nt phasiRNAs are shown in the first and the second sheet, respectively.
(XLSX)
