# Supplementary figures and images for: Genome-Wide Discovery and Analysis of Phased Small Interfering RNAs in Chinese Sacred Lotus
Source: PLoS One. 2014 Dec 3;9(12):e113790. doi: 10.1371/journal.pone.0113790 (PMC4254747; doi:10.1371/journal.pone.0113790)

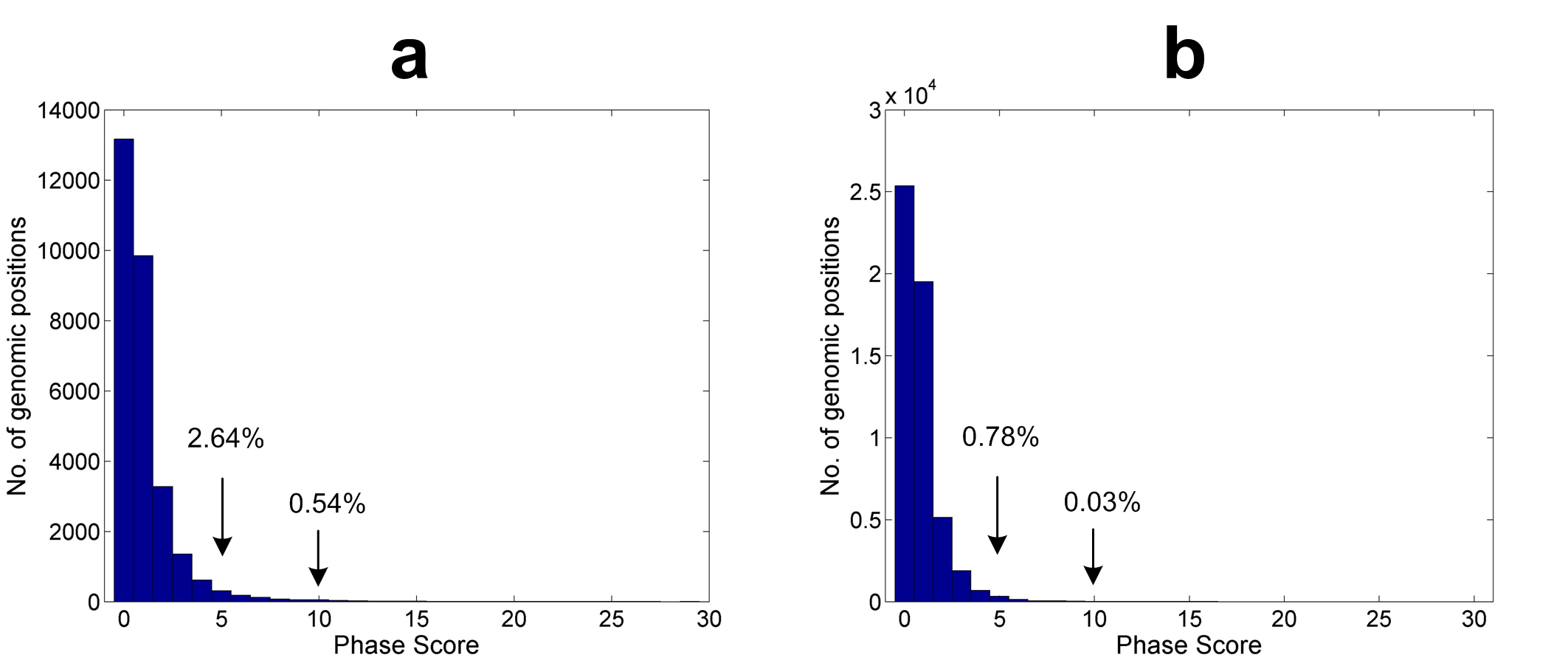

Supplement: Figure S1 — The histogram of Phase Scores. (a) The histogram of Phase Scores of 21 nt PHAS loci. (b) The histogram of Phase Scores of 24 nt PHAS loci. The percentage values above 5 and 10 are percentage of PHAS loci with Phase Scores larger than or equal to 5 and 10, respectively. (TIF) [file pone.0113790.s001.tif]

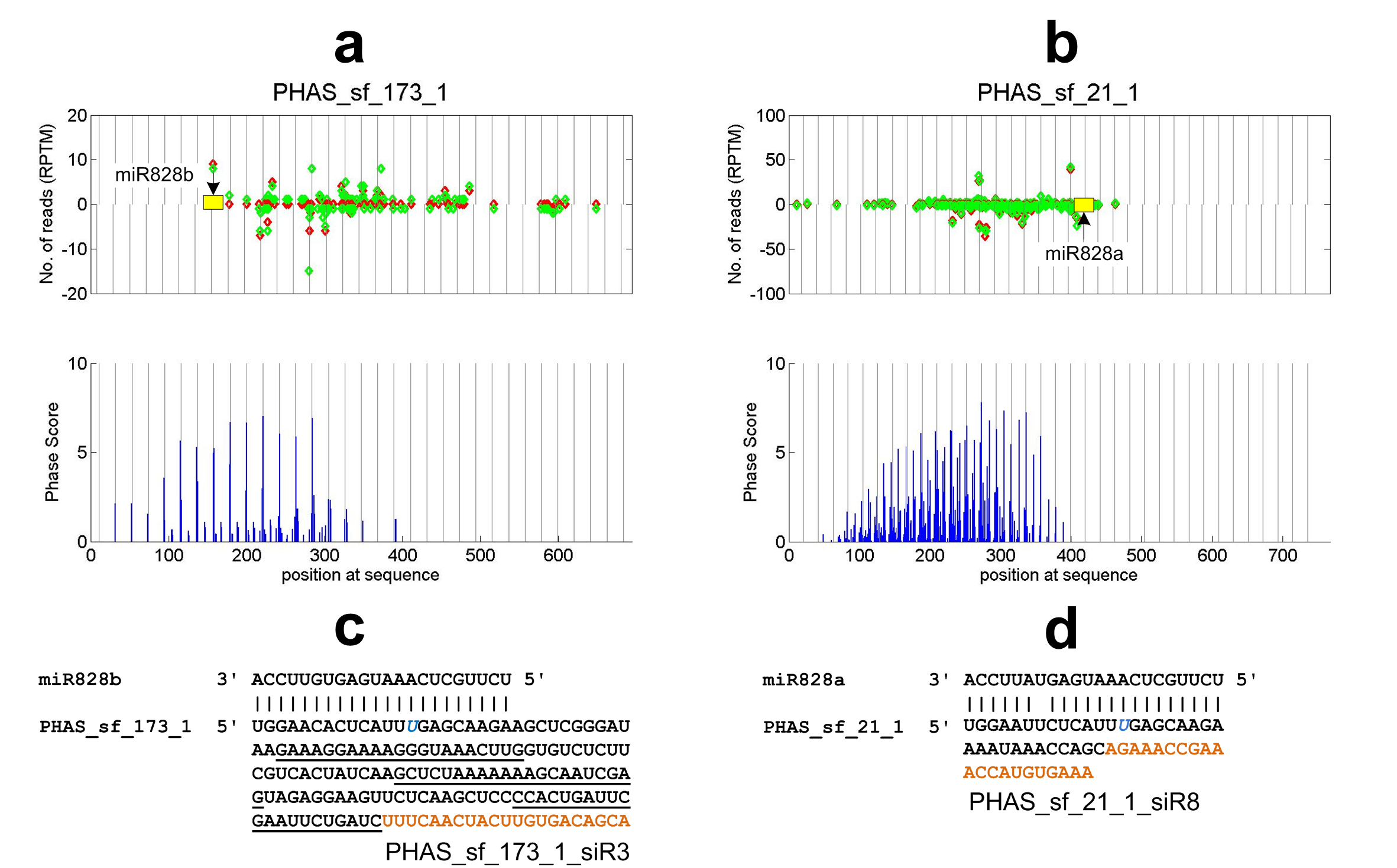

Supplement: Figure S2 — Two PHAS loci, PHAS_sf_173_1 and PHAS_sf_21_1, from putative MYB transcription factors. (a) - (b) The upper and lower panels show the read distribution and phase scores. The red and green diamonds represent the number of 21 nt reads, vertical axis, that appeared at the position of the PHAS loci, horizontal axis, in the flower and leaf small RNA libraries, respectively. The yellow boxes in the read distribution panel represent the miRNA complementary sites. Sites pointed by miRNAs from above and under zero read line means miRNAs complement to the plus and minus strand of the predicted PHAS loci, respectively. (c) - (d) The miR828b complementary site on PHAS_sf_173_1 and miR828a complementary site on PHAS_sf_21_1, respectively. The italic characters are the expected cleavage sites induced by miR828, i.e., the start positions of the phasiRNAs. The brown regions are phasiRNAs that appear in our sequencing libraries, PHAS_sf_173_1_siR3 (in Part c) and PHAS_sf_21_1_siR8 (in Part d), which are 126 nt (six 21 nt phases) and 21 nt (one 21 nt phase) downstream of the expected cleavage sites induced by miR828, respectively. (TIF) [file pone.0113790.s002.tif]

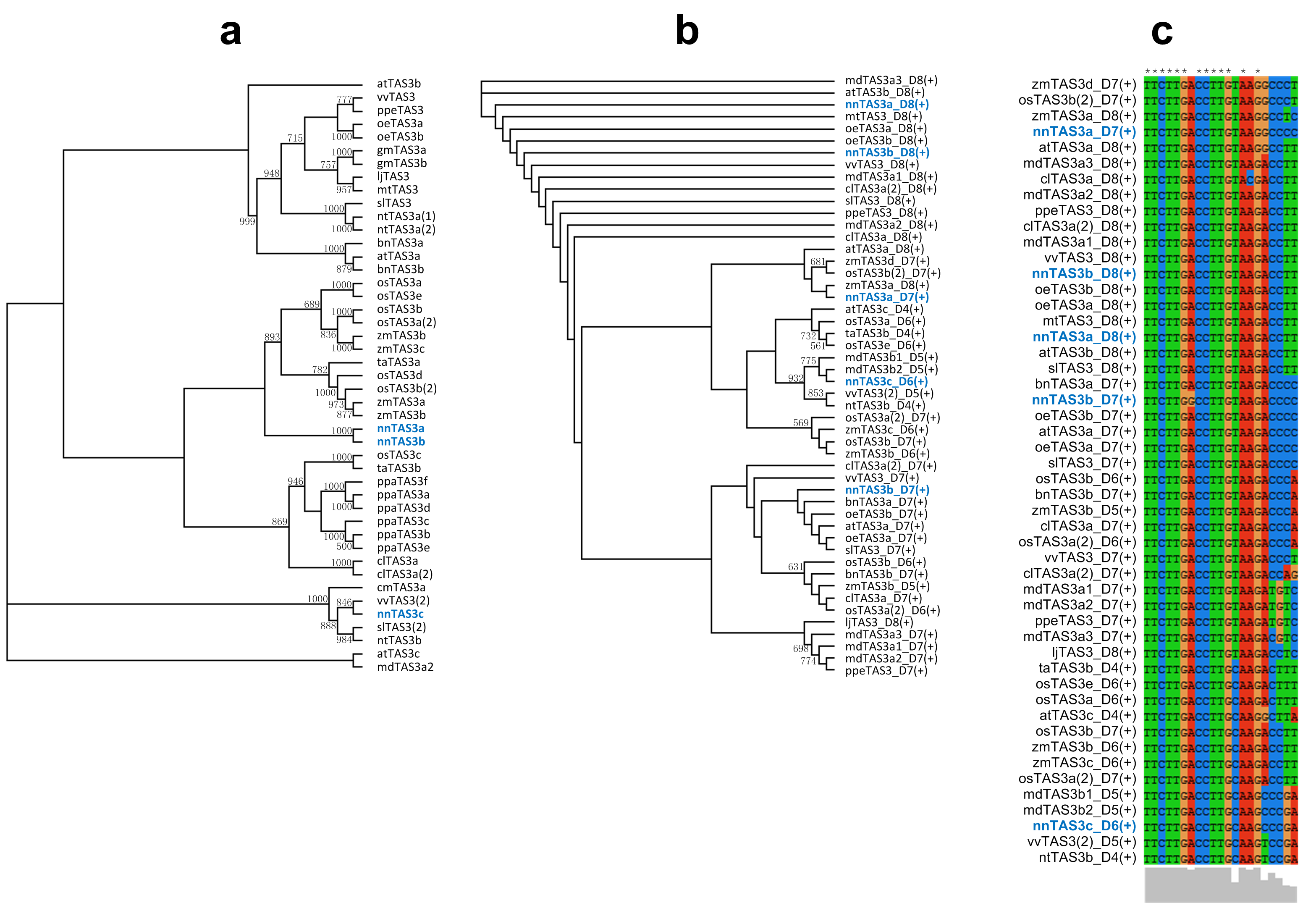

Supplement: Figure S3 — The conservation analysis of TAS3 loci and derived tasiRNAs in Chinese sacred lotus and other species. (a) The phylogenetic tree of TAS3. (b) The phylogenetic tree of TAS3 derived tasiRNAs. (c) The multiple sequence alignment of TAS3 derived tasiRNAs generated with ClustalX (version 2.1) [37]. The sequences of TAS3 loci and derived tasiRNAs were used to construct the phylogenetic trees with the Bootstrap Neighbor-Joining algorithm implemented in ClustalX (version 2.1). Then, the trees were visualized with TreeView [38]. The numbers in the trees are bootstrap values greater than 500 (50%). The lower case letters at the beginnings of the names of TAS3 and tasiRNAs stand for the species, i.e., at (Arabidopsis thaliana), bn (Brassica napus), cl (Cunninghamia lanceolata), cm (Cucumis melo), gm (Glycine max), lj (Lotus japonicus), md (Malus domestica), mt (Medicago truncatula), nn (Nelumbo nucifera (Gaertn)), nt (Nicotiana tabacum), oe (Olea europaea), os (Oryza sativa), ppa (Physcomitrella patens), ppe (Prunus persica), sl (Solanum lycopersicum), ta (Triticum aestivum), vv (Vitis vinifera), and zm (Zea mays). nnTAS3a, nnTAS3b and nnTAS3c are scaffold_106_1, scaffold_65_1, and scaffold_10_1, respectively. The tasiRNAs of TAS3a/b/c in Chinese sacred lotus are given in Figure 3d. (TIF) [file pone.0113790.s003.tif]

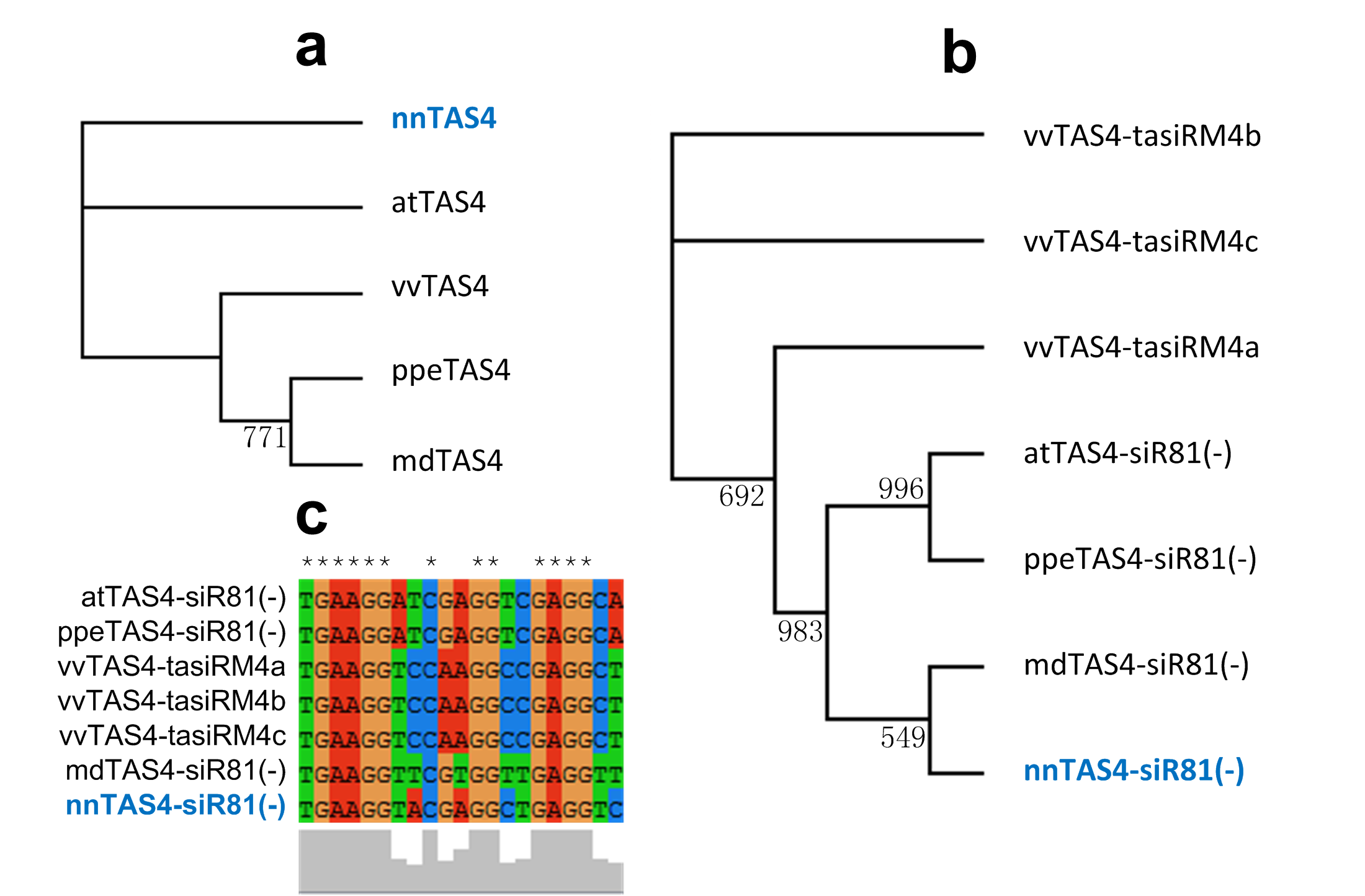

Supplement: Figure S4 — The conservation analysis of TAS4 loci and derived tasiRNAs in Chinese sacred lotus and other species. (a) The phylogenetic tree of TAS4. (b) The phylogenetic tree of TAS4 derived tasiRNAs. (c) The multiple sequence alignment of TAS4 derived tasiRNAs. The legend are the same as those of Figure S3. The lower case letters at the beginnings of the names of TAS3 and tasiRNAs stand for the species, i.e., at (Arabidopsis thaliana), md (Malus domestica), nn (Nelumbo nucifera (Gaertn)), ppe (Prunus persica), and vv (Vitis vinifera). nnTAS4 is sf_39_1 and nnTAS4-siR81(−) is sf_39_1_siR4. (TIF) [file pone.0113790.s004.tif]
